# Antifungal and Immunomodulatory Activities of Brazilian Savannah *Solanum lypocarpum* Tree-Associated Streptomyces Isolates

**DOI:** 10.3390/ph18081158

**Published:** 2025-08-05

**Authors:** Camila Bontempo Nunes, Kunal Ranjan, Fernando Pacheco Rodrigues, Marjorie de Carvalho Vieira Queiroz, Clara Luna Freitas Marina, Luis Alexandre Muehlmann, Anamélia Lorenzetti Bocca, Marcio José Poças-Fonseca

**Affiliations:** 1 Graduation Program in Animal Biology, University of Brasilia, Brasilia 70910-900, Brazil; c.bontn@gmail.com (C.B.N.); mpossas@unb.br (M.J.P.-F.); 2 Department of Genetics and Morphology, University of Brasilia, Brasilia 70910-900, Brazil; fprodrigues@unb.br; 3 Amity Institute of Biotechnology, Amity University Jharkhand, Ranchi 835303, India; kukkukr.ranjan@gmail.com; 4 Graduation Program in Molecular Pathology, Faculty of Medicine, University of Brasilia, Brasilia 70910-900, Brazil; 5 School of Health Sciences and Technologies, Campus Ceilandia, University of Brasilia, Brasilia 72220-275, Brazil; marjoriecvq94@gmail.com; 6 Laboratory of Applied Immunology, Department of Cellular Biology, Institute of Biological Sciences, University of Brasilia, Brasilia 70910-000, Brazil; claraluna93@gmail.com (C.L.F.M.); albocca@unb.br (A.L.B.); 7 Bi-Institutional Translational Medicine Platform, Oswaldo Cruz Foundation (Fiocruz), Ribeirão Preto 14049-900, Brazil

**Keywords:** Brazilian Central Savannah (Cerrado), *Solanum lycocarpun*, actinobacteria, antifungal activity, *Cryptococcus* spp., *Candida* spp., immunomodulation

## Abstract

**Background/Objectives:** Actinobacteria are one of the largest bacterial phyla. These microbes produce bioactive compounds, such as antifungals, antibiotics, immunological modulators, and anti-tumor agents. Studies on actinobacteria isolated from the Brazilian Savannah biome (Cerrado) are scarce and mostly address metagenomics or the search for hydrolytic enzyme-producing microbes. *Solanum lycocarpum* (lobeira) is a tree widely employed in regional gastronomy and pharmacopeia in Central Brazil. **Methods:** In this work, 60 actinobacteria isolates were purified from the rhizosphere of *S. lycocarpum*. Eight *Streptomyces* spp. isolates were selected for in vitro antifungal activity against *Cryptococcus neoformans* H99, the *C. neoformans* 89-610 fluconazole-tolerant strain, *C. gattii* NIH198, *Candida albicans*, *C. glabrata,* and *C. parapsilosis*. The ability of the aqueous extracts of the isolates to induce the in vitro secretion of tumor necrosis factor (TNF-α), nitric oxide (NO), interleukin-6 (IL-6), and IL-10 by murine macrophages was also evaluated. **Results:** All extracts showed antifungal activity against at least two yeast species. *Streptomyces* spp. LAP11, LDB2, and LDB17 inhibited *C. neoformans* growth by 40–93%. Most extracts (except LAP2) also inhibited *C. gattii*. None inhibited *C. albicans*, but all inhibited *C. glabrata* (40–90%). *Streptomyces* sp. LAP8 extract increased nitric oxide production by approximately 347-fold in murine macrophages, while LDB11 extract suppressed LPS-induced TNF-α production by 70% and simultaneously increased IL-10 secretion, suggesting immunosuppressive potential. **Conclusions:** The results revealed that Cerrado actinobacteria-derived aqueous extracts are potential sources of antifungal and immunomodulatory biocompounds.

## 1. Introduction

Invasive fungal infections (IFIs) are mainly caused by *Candida*, *Aspergillus*, and *Cryptococcus* species, representing a major clinical concern in morbidity and mortality [1,2,3]. According to data from the Leading International Fungal Education (LIFE) initiative, about a billion people are infected, and 1.5 million deaths are reported yearly [4,5]. Based on global data from 2019 to 2021, approximately 6.5 million people develop invasive fungal infections each year, resulting in around 2.5 million deaths [6]. Limited therapeutic agents, such as azoles, polyenes, and echinocandins, are available for treating IFIs [2,5,7]. The selection of clinically resistant strains poses a severe threat to public health. The restrained pipeline for novel antifungal agents, the increasing number of immunosuppressed individuals, and the plasticity of the virulence phenotypes demand the urgent development of new antifungal drugs [8].

The Actinobacteria phylum is the original source of approximately 70% of the antibiotics currently known, with the genus *Streptomyces* being responsible for half of the compounds under clinical trials [9,10]. Studies on the antifungal activity of actinobacteria against the IFIs causing microbes, particularly *Cryptococcus*, are limited [11,12,13,14,15,16]. Actinobacteria-derived metabolites represent a potential source for drug screening and/or design targeting multi-resistant microorganisms. As Barka [17] pointed out, actinobacteria metabolites can also be employed in immunomodulatory therapies. Previously, bioactive compounds such as octacosane, hexatriacontane, heptacosane, tetratetracontane, myristic Acid vinyl ester, and Bad368.1 from actinobacteria demonstrated antimicrobial as well as immunomodulatory properties [18,19,20]. This research illustrates the dual potential of actinobacterial metabolites in both antimicrobial and immunomodulatory applications, which supports the rationale for investigating Brazilian savannah-derived *Streptomyces* in this context.

Inflammation is a mammalian protective immunological response to external stimuli, such as infections or tissue damage. Several mediators control the inflammatory reactions, including nitric oxide (NO), prostaglandin E2, interleukin-1 (IL-1), IL-6, IL-10, and tumor necrosis factor (TNF-α) [21,22]. The current immunomodulatory drugs available for clinical use can present significant negative side effects, such as nephrotoxicity and neurotoxicity [4,23,24]. In this view, the bioprospection of new immunomodulatory compounds should be encouraged to improve clinical immunotherapy and reduce adverse effects.

The Cerrado is the second-largest biome in Brazil. It corresponds to savanna-like vegetation and represents a global biodiversity hotspot, with over 4800 plant species [25]. Lobeira (*Solanum lycocarpum*) is a small tree within the Solanaceae family that is native to South America and predominantly found in the Brazilian Cerrado. It is known for being resistant and adaptable to dry and hot environments. Parts of these plants are employed by local people as anti-inflammatory, antioxidant, and cholesterol level-controlling agents. They are also considered for treating diabetes and obesity [26,27,28]. Lobeira fruits correspond to about 30% of the *lobo-guará* (*Chrysocyon brachyurus*) diet [29]. Nonetheless, this tree, or its associated microorganisms, has not been explored for biotechnological applications.

This work aimed to screen and isolate actinobacteria from the rhizosphere of *S. lycocarpum* (*lobeira*) from the Cerrado biome and to assess their potential as producers of antifungal and immunomodulatory molecules. This article is based on the thesis authored by Nunes [30].

Our study unraveled lobeira-associated *Streptomyces* spp. isolates whose aqueous extracts presented growth inhibition properties against *Cryptococcus neoformans* H99, the *C. neoformans* 89-610 fluconazole-tolerant strain, *C. gattii* NIH198, *Candida albicans*, *C. glabrata*, and *C. parapsilosis*. Pro- and anti-inflammatory modulation activities (TNF-α, Nitric oxide, IL-6, and IL-10) on murine bone marrow-derived macrophages (BMDMs) in vitro were also described.

## 2. Results

### 2.1. Macroscopic and Microscopic Features of the Actinobacteria Isolates

The collected soil samples yielded about 60 isolates. The capacity to produce pigments could point to actinobacteria that produce bioactive compounds having antimicrobial activity [31,32,33]. Thirteen isolates that produced soluble pigment and/or presented mycelium coloration were selected for further investigation.

These isolates were subcultured onto Asparagine Glycerol Agar (AGA) medium, and purity was confirmed by microscopic analysis of liquid cultures grown for 5 days in rich non-selective medium. Table 1 summarizes the macroscopic characteristics of the actinobacteria isolates. All the isolates produced soluble pigments except LDB 32 and LDB 34, which presented blue and pink mycelia, respectively. LDB 17 and LS3 produced yellow soluble pigments and presented white mycelium. LS did not produce pigmented aerial mycelium or spores.

**Figure A1:**
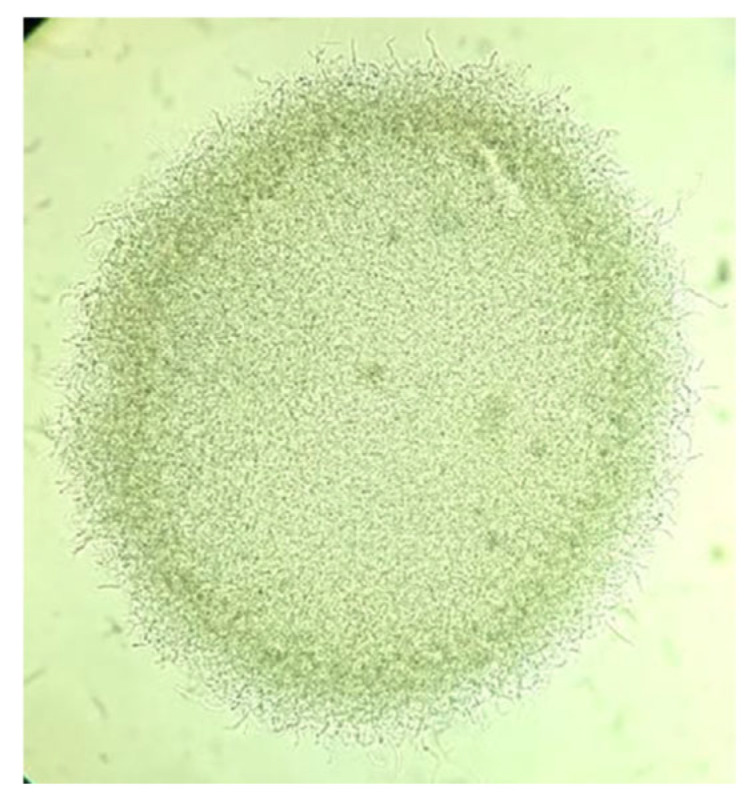
Optical microscopy of actinobacteria isolates: Pellet of the LAP8 isolate, under a 40× objective.

**Figure A2:**
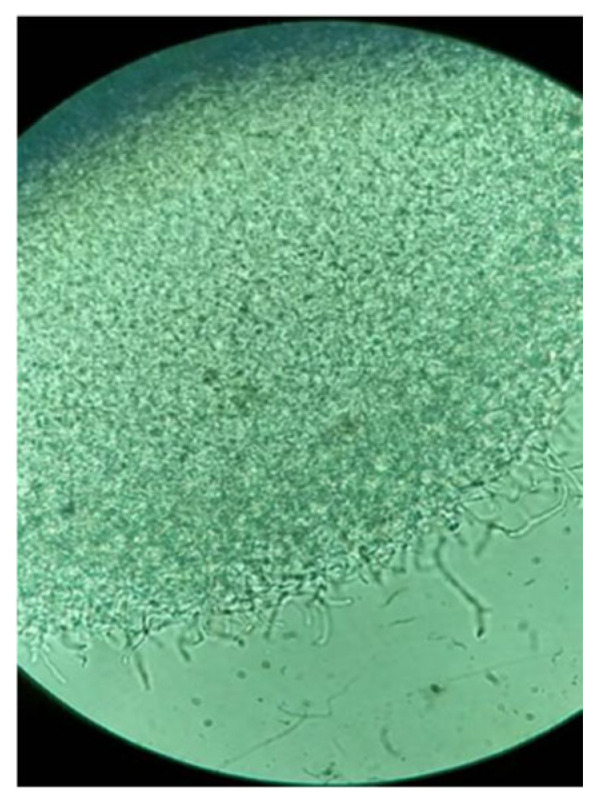
Optical microscopy of actinobacteria isolates: LAP8 isolate, under 100× objective.

**Figure A3:**
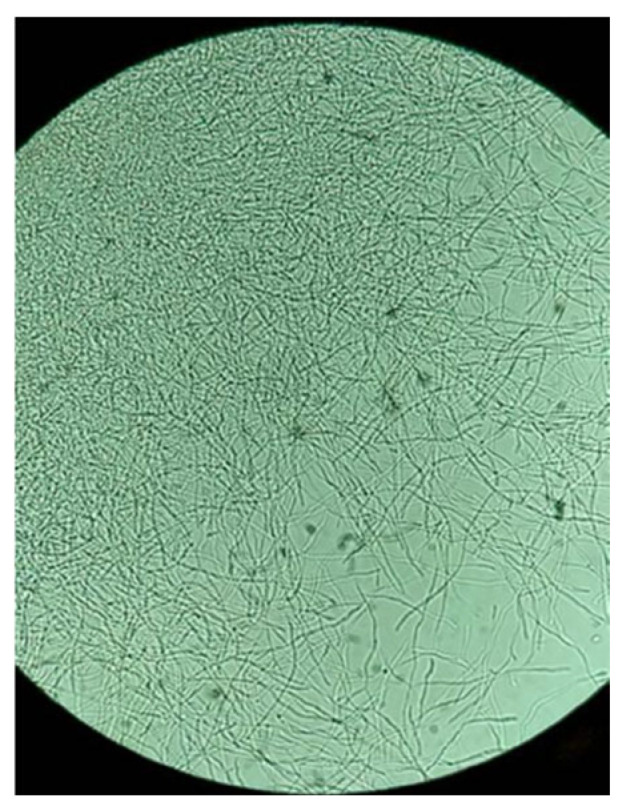
Optical microscopy of actinobacteria isolates: LAP11 Isolate, under a 100× objective.

Microscopically, all isolates formed branched hyphae in solid and liquid cultures. The hyphae of the isolates were dense in the center and more spaced towards the margins of the colonies (Appendix A, Figure A1, Figure A2 and Figure A3).

### 2.2. Actinobacteria Identification and Phylogenetic Analysis

We successfully amplified and sequenced the 16S rDNA region for 10 selected isolates. When comparing our sequences with those of GenBank, all the isolates were identified as belonging to the genus *Streptomyces*. Except for the isolates LAP3 and LAP11, which showed a greater similarity to *S. luteogriseulus* (98.85% of similarity) and *S. roseogriseus* (98.85% of similarity), respectively, all the others presented similarity to more than two species of *Streptomyces*.

**Figure 1:**
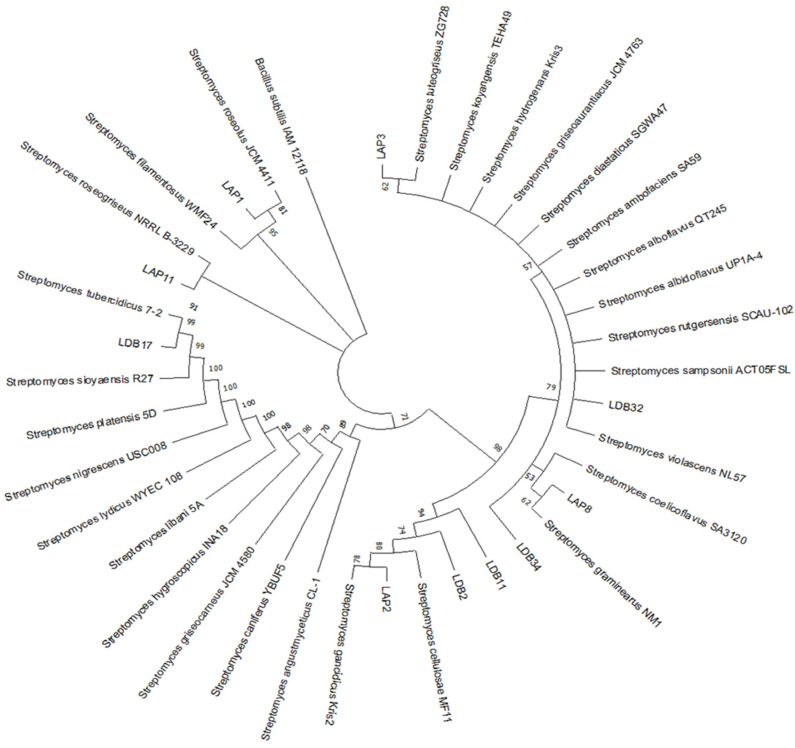
Phylogenetic tree of actinobacteria isolates based on 16S rDNA sequences. The evolutionary relationship was made by the maximum likelihood method and results from bootstrap statistical analysis. Values next to nodes indicate bootstrap support (%) based on analysis of 1000 replicas. The branches referring to support values lower than 50% were condensed.

Although using the 16S rDNA alone is insufficient to establish species-level identification of *Streptomyces*, we performed a phylogenetic analysis using this region to identify the closest evolutionary relationships with the sequences obtained from GenBank (Figure 1). LAP1 was identified as more closely related to *S. roseolus*, LAP2 to *S. gancidicus*, LAP8 to *S. graminearus*, LDB2 to *S. cellulosae*, and LDB17 to *S. tubercidicus*. Isolate LDB11 showed greater evolutionary proximity to isolate LDB2. It was not possible to approximate the relationship of LDB32 and LDB34 with the other sequences used in this analysis; therefore, these isolates were excluded from further studies.

### 2.3. Accession Numbers of the Actinobacteria Isolate Nucleotide Sequences

The nucleotide sequences of the actinobacteria isolates were submitted to the NCBI database. The following accession numbers were assigned: OR816059 for LAP1, OR816061 for LAP2, OR816062 for LAP3, OR816063 for LAP8, OR816060 for LAP11, OR816064 for LDB11, OR816065 for LDB17, OR816066 for LDB2, OR816067 for LDB32, and OR816068 for LDB34.

### 2.4. Antifungal Activity of the Streptomyces spp. Extracts

**Figure 2:**
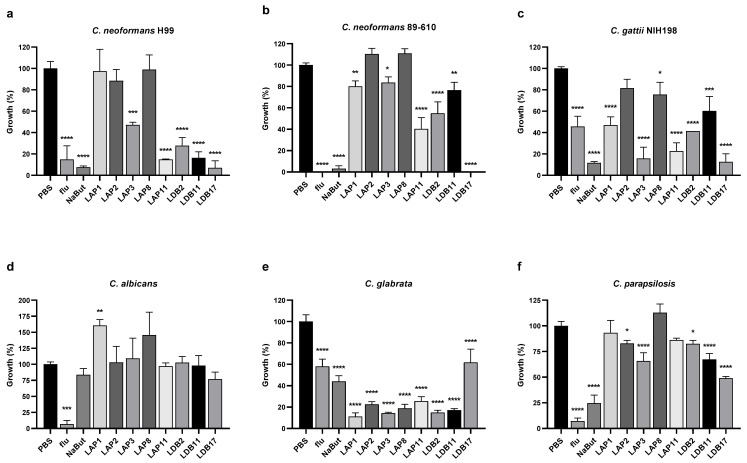
Growth inhibition by actinobacteria extracts at a final concentration of 10% (*v*/*v*) in RPMI-1640 media. *Cryptococcus* strains: (**a**) *C. neoformans* H99, (**b**) *C. neoformans* 89-610, and (**c**) *C. gattii* NIH198; *Candida* strains: (**d**) *C. albicans*, (**e**) *C. glabrata*, and (**f**) *C. parapsilosis*. Yeasts were incubated with extracts for 72 h at 37°C in 96-well plates. PBS corresponds to positive growth control. Flu (fluconazole) and NaBut (sodium butyrate) were the control drugs. Statistical analysis was performed by one-way ANOVA followed by Turkey’s post hoc test, in multiple comparisons of group tests to the control (PBS), with a significant difference indicated relative to the control group; * *p* < 0.05, ** *p* < 0.01, *** *p* < 0.001, and **** *p* < 0.0001 compared to the control group. This data represents the result obtained from three independent experimental replicates, *n* = 3.

The antifungal activity of resuspended crude extracts of eight *Streptomyces* isolates was evaluated against *C. neoformans* H99, *C. neoformans* 89-610, *C. gattii* NIH198, *C. albicans*, *C. glabrata,* and *C. parapsilosis* (Figure 2).

The extract of *Streptomyces* sp. LDB17 was the most effective against *C. neoformans* H99 growth (93% inhibition), followed by *Streptomyces* sp. LAP11 (85%), *Streptomyces* sp. LDB11 (83%), *Streptomyces* sp. LDB 2 (72%), and *Streptomyces* sp. LAP3 (53%) extracts. The extracts of *Streptomyces* spp. such as LAP1, LAP2, and LAP8 did not reduce *C. neoformans* H99 growth (Figure 2a).

Considering the fluconazole-resistant *C. neoformans* 89-610 strain, growth was completely abolished by the LDB17 extract. LAP11 and LDB2 extracts inhibited growth by 50–60%, and LAP1, LAP3, and LDB11 extracts inhibition ranged from 15 to 25%. LAP2 and LAP8 did not affect the growth of *C. neoformans* 89-610 (Figure 2b).

The *Streptomyces* sp. LDB17 extract was also the most effective against the growth of *C. gattii* NIH198 (87%), followed by LAP3 (84%), LAP11 (77%), LDB2 (59%), LAP1 (53%), LDB11 (40%), and LAP8 (24%). The *Streptomyces* sp. LAP2 extract did not reduce the growth of *C. gattii* (Figure 2c).

None of the extracts significantly reduced the growth of *C. albicans*. Curiously, *Streptomyces* sp. LAP1 extract significantly increased *C. albicans* growth (Figure 2d). In contrast, all the extracts significantly inhibited the growth of *C. glabrata* from 75 to 85%, except for *Streptomyces* sp. LDB17 (38%). Interestingly, *C. glabrata* was more sensitive to all the extracts than the controls exposed to fluconazole or sodium butyrate, except LDB17 (Figure 2e).

Extracts of *Streptomyces* spp isolates LAP2, LAP3, LDB2, LDB11, and LDB17 significantly inhibited the growth of *C. parapsilosis*. However, the activity of these extracts was more subtle (17–34% inhibition) than what was observed for *C. glabrata*, except for *Streptomyces* sp. LDB17 (50%) (Figure 2f).

Overall, *Cryptococcus* was more susceptible to the *Streptomyces* crude extracts than *Candida*, particularly the *Streptomyces* sp. LDB17 extract. All the extracts presented antifungal activity against at least two different microorganisms.

### 2.5. Cytotoxicity to Murine BMDMs of the Streptomyces spp. Extracts

**Figure A4:**
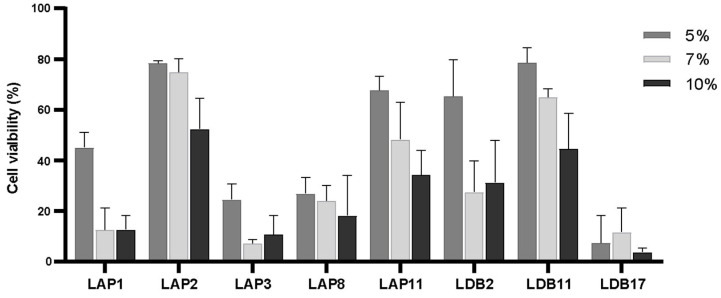
Cytotoxicity assay in murine macrophages under interaction for 24 h with actinomycetes extracts at final concentrations of 5%, 7%, and 10%.

Initially, three different concentrations of extracts (5%, 7%, and 10% of the final volume) were assayed with BMDM cells (Appendix A, Figure A4). All the extracts demonstrated high cytotoxicity at a final concentration of 10%. *Streptomyces* sp. LDB17 extract was the most cytotoxic (50% of cell viability), while LAP2 extract was the least (approximately 5%). The final extract concentration of 7% also exhibited high cytotoxicity to murine BMDMs. Only the LAP2, LAP11, and LDB11 extracts compromised BMDM cell viability below 50%. On the other hand, *Streptomyces* spp. LAP1, LAP3, and LDB17 extracts presented cytotoxicity rates at the 10% concentration.

The BMDMs were incubated with 5% of the *Streptomyces* spp. extracts, which revealed LAP2 and LDB11 as the least cytotoxic (approximately 78% of cell viability), followed by *S*. LAP11 (approximately 70%) and LDB2 (65%). The other extracts resulted in less than 50% cell viability. The exposure to the *Streptomyces* sp. LDB17 extract resulted in only 8% of cell viability (Appendix A, Figure A4). Considering the minimum cell viability value of 70% for the subsequent experiments, the extract concentrations were 1% (LAP1, LAP3, LAP8, LDB2, and LDB17) and 5% (LAP2, LAP11, and LDB11).

**Figure 3:**
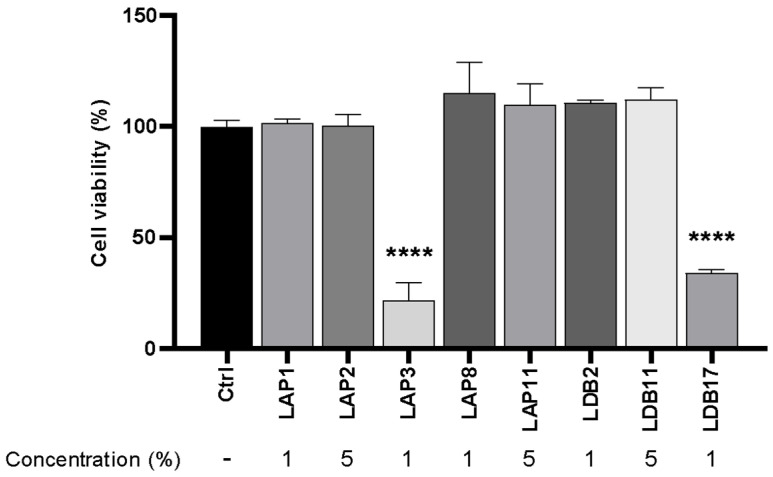
Cytotoxicity assay of *Streptomyces* extracts in M1-like macrophages under concentrations of actinobacteria extracts initially defined for experiments. The control group (ctrl) corresponded to 100% cell viability without treatment. Statistical analysis was performed by two-way ANOVA on multiple comparisons of test-to-control (PBS) groups, with a significant difference indicated relative to the control group; **** *p* < 0.0001 compared to the control group. Representative graph from 3 independent experiments, *n* = 3.

Cytotoxicity assays were then performed with the extract at the abovementioned concentrations (Figure 3). The extract concentrations for the subsequent experiments were defined as 5% (LAP1, LAP8, and LDB2) and 0.5% for *Streptomyces* spp. LAP3 and LDB17, since these concentrations would not induce immune cell reactions related to cytotoxicity.

### 2.6. TNF-α Production by Murine BMDMs Exposed to Streptomyces spp. Extracts

**Figure 4:**
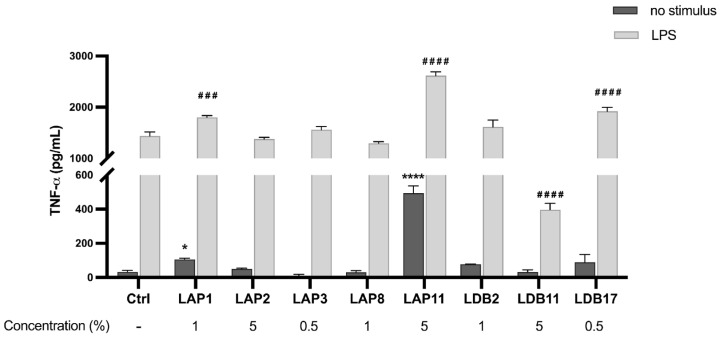
Quantifying TNF-α in the supernatant of M1-like macrophages exposed to *Streptomyces* extracts for 24 h. Statistical analysis was performed by two-way ANOVA, for which (*) indicated a significant difference compared to the control group within the “Without stimulus” group, and (#) indicated a significant difference compared to the control group within the “LPS” group. The statistical difference was demonstrated with * *p* < 0.05 and **** *p* < 0.0001 compared to the unstimulated control group; ### *p* < 0.001 and #### *p* < 0.0001 compared to the LPS-stimulated control group. Representative graph from 3 independent experiments, *n* = 3.

The ability of the extracts to induce the production of TNF-α was evaluated (Figure 4). *Streptomyces* spp. LAP1 and LAP11 extracts significantly induced TNF-α production by BMDMs (*p* < 0.001 and *p* < 0.0001, respectively). The *Streptomyces* sp. LDB17 extract did not present a statistical difference from the group not stimulated with LPS. Nonetheless, these three extracts showed an additive action to the stimulus with LPS. The LDB11 extract significantly inhibited the LPS-induced TNF-α production (70%) compared to the control group stimulated with LPS (*p* < 0.001) (Figure 4).

### 2.7. Nitric Oxide Production by Murine BMDMs Incubated with Streptomyces spp. Extracts

**Figure 5:**
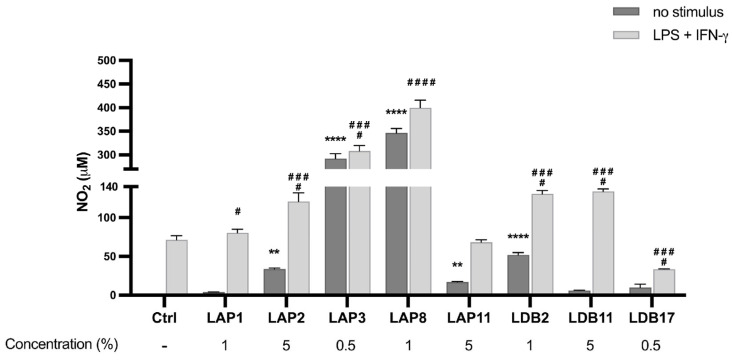
Quantifying nitric oxide (NO) in the supernatant of M1-like macrophages exposed to *Streptomyces* extracts for 24 h. Statistical analysis performed by two-way ANOVA, for which the asterisk indicates a significant difference compared to the control group within the “Without stimulus” group, and the hashtag indicates a significant difference compared to the control group within the “LPS” group + IFN-γ. The statistical difference was demonstrated with ** *p* < 0.01 and **** *p* < 0.0001 compared to the unstimulated control group; # *p* < 0.05, ### *p* < 0.001, and #### *p* < 0.0001 compared to the LPS-stimulated control group. Representative graph from 3 independent experiments, *n* = 3.

The *Streptomyces* sp. LAP8 extract augmented the NO levels by 347 times when compared to the control group without any stimulus (*p* < 0.001), followed by LAP3 (292x), LDB2 (52x), LAP2 (34x), and LAP11 (17x) (Figure 5). The *Streptomyces* spp. isolates of LAP1, LDB11, or LDB17 extracts did not induce NO production. When BMDMs were stimulated by LPS and IFN-γ, LAP8 extract increased the NO production by 4.5 times, compared to the control group (*p* < 0.0001), followed by LAP3 (3x) (*p* < 0.001). Only the LDB17 extract presented a suppressive effect on NO production (53%) (*p* < 0.001). The LAP11 extract did not interfere with NO production upon stimulation (Figure 5).

Based on the cytotoxicity assays, TNF-α, and NO production data, the extracts of *Streptomyces* spp. LAP1 and LAP11 (TNF-α inducers) and *Streptomyces* spp. LAP3 and LDB17 (high cytotoxicity) were excluded from further experimental analysis. The *Streptomyces* spp. LAP2, LAP8, LDB2, and LDB11 extracts were then selected for the in vitro assays of the macrophage microbicidal activity.

### 2.8. Fungal Burden Inside Murine BMDMs

**Figure 6:**
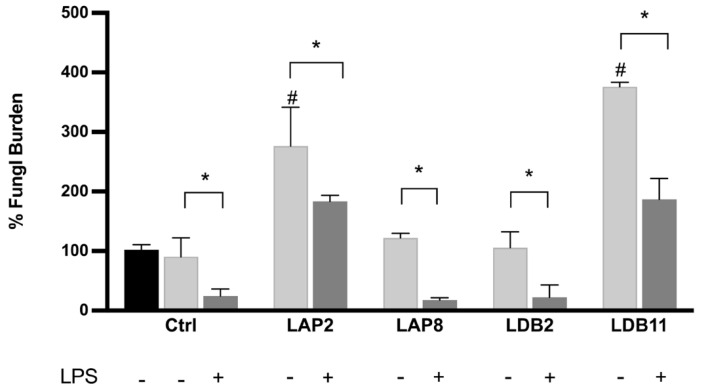
Fungal burden inside the macrophage. *C. neoformans* H99 CFU counts after 2 h (black bar) and 24 h for macrophages non-treated (CTLR) or treated with LAP2, LAP8, LDB2, and LDB11 extracts. The group with and without activation by LPS was formed. In the 2 h group without treatment, LPS was performed for fungus internalization analysis, which was considered the 100% fungal burden. Statistical analysis was performed by two-way ANOVA, where the asterisk indicates a significant difference compared to the control group, within the “Without stimulus (24 h)” group or within the “LPS (24 h)” group, and the hashtag indicates a significant difference between the 24 h groups with and without stimulus (# *p* < 0.05), for the same extract. The statistical difference was demonstrated with * *p* < 0.05 compared to the LPS-stimulated control group. Representative graph from 3 independent experiments, *n* = 3.

BMDMs were incubated with *C. neoformans* H99 in the absence or the presence of the extracts of *Streptomyces* spp. LAP2, LAP8, LDB2, or LDB11 to assess whether the extracts could modulate the microbicidal activity of these cells (Figure 6).

*Streptomyces* spp. LAP8 and LDB2 extracts did not show a significant difference in the fungal load retrieved from BMDMs compared to the controls, stimulated or not with LPS. These extracts did not interfere with fungus proliferation within the macrophage or the ability of macrophages to kill internalized cells after activation with LPS.

The BMDMs stimulated with LAP2 and LDB11 extracts, in the presence or not of LPS, presented a higher fungal load than the control group at 24 h (*p* < 0.05). In this view, the proliferation rate of internalized *C. neoformans* H99 yeasts was higher under exposure to the extracts, despite both inducing higher levels of NO when also stimulated with LPS.

### 2.9. Quantification of Cytokines After the Infection of BMDMs with C. Neoformans H99

TNF-α production in the supernatant of the BMDMs infected with yeast cells was evaluated. IL-6 and IL-10 production analysis was included to better assess the activation of the inflammatory response induced by the *Streptomyces* extracts.

**Figure 7:**
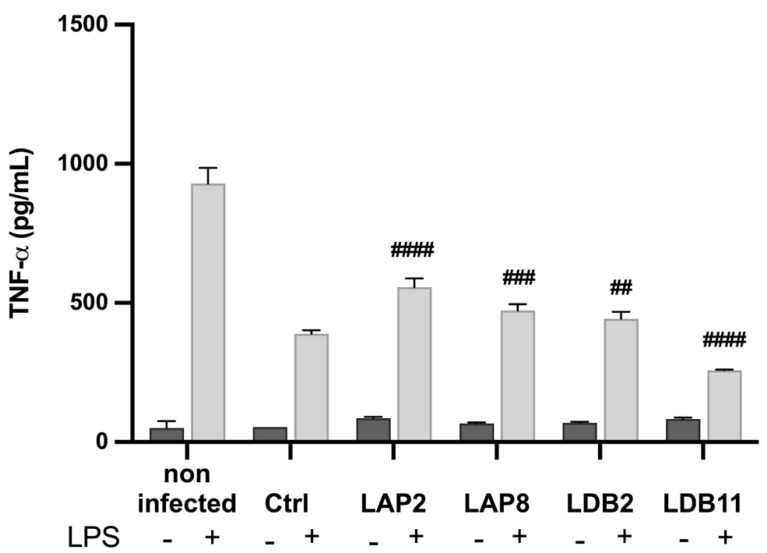
Quantifying TNF-α in the supernatant of macrophages infected by H99 and treated with the extracts, after 24 h of incubation. Quantification was carried out by the ELISA method. Statistical analysis was performed by one-way ANOVA. LPS induced a significantly higher production of TNF-α in all groups (*p* < 0.001). The hashtags indicate statistically significant differences between the experimental group vs control, in the LPS-stimulated condition, with ## for *p* < 0.01; ### for *p* < 0.001; and #### for *p* < 0.0001. This data represents the results obtained from three independent experimental replicates.

The BMDMs activated with LPS and infected with H99 showed an increase in the production of TNF-α when exposed to the extracts of *Streptomyces* spp. LAP2 (*p* < 0.001), LAP8 (*p* < 0.01), and LDB2 (*p* < 0.05). A decreased TNF-α production was observed upon exposure to the LDB11 extract (*p* < 0.001) (Figure 7). There was no significant difference in the production of TNF-α in unstimulated and infected BMDMs exposed to the extracts.

**Figure 8:**
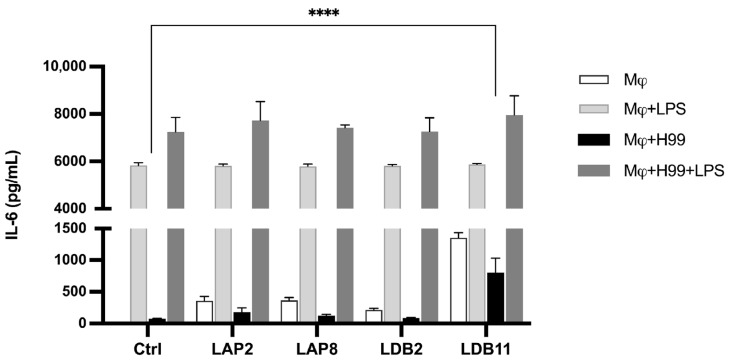
IL-6 concentration in macrophage supernatant with or without fungal infection after interaction with extracts for 24 h. Quantification was carried out by the ELISA method. Statistical analysis was performed by one-way ANOVA. LPS induced a significantly higher production of IL-6 in all groups (*p* < 0.001). **** for *p* < 0.0001. This data represents the result obtained from three independent experimental replicates.

The influence of the extracts on the IL-6 production by BMDMs not infected with yeasts was initially analyzed (Figure 8).

Without LPS, all the extracts induced significantly higher IL-6 production than the macrophages alone (*p* < 0.001). The *Streptomyces* sp. LDB11 extract was the most effective inducer compared to the control group, which was not treated with the extract. In contrast, no significant difference was observed in the IL-6 production between the groups stimulated with LPS and the control.

Subsequently, the production of IL-6 in the supernatant of BMDMs infected by *C. neoformans* H99 was evaluated. Similar to the previous experiment, with the LPS stimulus, there was no significant difference in IL-6 production in the presence of the extracts compared to the controls. The *Streptomyces* sp. LDB11 extract induced IL-6 production by BMDMs not activated by LPS but infected with H99 (Figure 8).

The extracts were also analyzed to produce IL-10. The *Streptomyces* sp. LDB11 extract induced IL-10 production when the BMDMs were stimulated with LPS, where TNF-α production was inhibited. This effect was not observed in the cells treated with isolates LAP2, LAP8 and LDB2, neither in groups non stimulated with LPS. 

**Figure 9:**
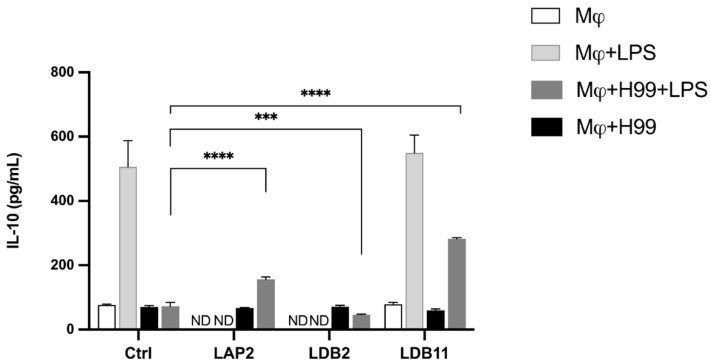
Quantifying IL-10 in the supernatant of macrophages infected by H99 or non-infected and treated or not with the extracts, after 24 h of incubation. Quantification was carried out by the ELISA method. Statistical analysis was performed using two-way ANOVA and *t*-test, respectively, for which the asterisk indicates a significant difference. The statistical difference was demonstrated by *** *p* < 0.001 and **** *p* < 0.0001 compared to the H99-infected and LPS-stimulated control group. Representative graph from 3 independent experiments, *n* = 3.

After that, the analysis of IL-10 production by BMDMs infected with *C. neoformans* H99 and exposed to the isolates LAP2, LDB11, and LDB2 extracts was also conducted. Compared to the positive control, the induction of IL-10 was observed in infected cells activated with LPS and incubated with *Streptomyces* spp. LAP2 and LDB11 extracts (*p* < 0.001 and *p* < 0.0001, respectively), which corroborate their anti-inflammatory properties. The LDB11 extract was the strongest inducer compared with the other extracts (Figure 9). LPS-activated and LDB2 extract-exposed BMDMs showed a small decrease in IL-10 production. No significant difference was observed in the production of this cytokine between the BMDMs infected and not stimulated with LPS but exposed to the extracts and the control.

## 3. Discussion

Despite their ubiquitous distribution, most of the members of the actinobacteria phylum represent soil-dwelling microorganisms. Conditions such as temperature, pH, organic matter, or moisture content can influence the abundance and diversity of actinobacteria in the soil [34,35]. In soil, *Streptomyces* is the genus of actinobacteria most identified [9,10,17].

Studies on actinobacteria from the Cerrado soil are limited. Quirino [36] compared the bacterial community of a native Cerrado area (*Senso stricto*) and pastureland. Alfa-proteobacteria were most abundant in the *Senso stricto* area (26.4%), followed by acidobacteria (22.2%) and actinobacteria (19.4%). Actinobacteria were the most abundant in the pasture area (34.3%). Ferreira de Araujo and collaborators [37] described the bacterial diversity of the Cerrado biome in the Sete Cidades National Park, an Ecological Reserve in Northeast Brazil, where actinobacteria accounted for 21% of the total bacterial community. Conceição de Souza and Procópio [38] evaluated how environmental factors influenced the microbial communities in Cerrado. The authors demonstrated that actinobacteria were the most predominant phylum in drought and fire-burnt areas. Still, Proteobacteria and Firmicutes were most prevalent after the first rain and during the rainy season. Cavalcante et al. [39] recently also studied actinobacterial communities in Cerrado soils under different land uses. Actinobacteria were dominant (45.5–70.4%), with higher richness in agricultural soils but greater diversity and rare taxa in preserved areas. Up to 86% of genera in forest soils were unidentified, indicating high unexplored diversity. Rare orders like Rubrobacterales appeared only in agricultural sites. The study highlights the unseen impacts of human footprints, showing that agricultural land use reduces actinobacterial diversity and stability. At the same time, preserved Cerrado areas retain rare and uncatalogued taxa vital for ecosystem resilience.

In this work, about 60 actinobacteria isolates were obtained from the rhizosphere of *S. lycocarpum* (lobeira) in the Cerrado area of Distrito Federal, Brazil. There was no previous study on actinobacteria derived from the rhizosphere of Cerrado plants.

All the isolates were identified as *Streptomyces* spp. (Figure 1). This observation corroborates previous results that reported Actinobacteria, particularly *Streptomyces*, as the dominant phylum in Cerrado soil [36,40].

Invasive fungal infections due to *Cryptococcus*, *Candida,* and *Aspergillus* are major concerns worldwide [2,6,41,42,43]. *Cryptococcus* is an opportunistic yeast pathogen. Cryptococcal meningitis is estimated to affect over 223,000 people yearly [44]. *Candida albicans* is the most common cause of bloodstream infections, accounting for 43.4 to 56.9% of all cases worldwide. *C. glabrata* is the second most common species in North America (23.5%) and Europe (15.7%), Latin America (25.6%), and in the Asia Pacific area (13.7%) [2,43].

Actinobacteria, particularly *Streptomyces*, are notorious producers of biocompounds. In this view, new actinobacteria-derived metabolites should be explored as an alternative approach to combat drug-resistant fungal infections.

Recent research has emphasized the need for new agents with novel mechanisms of action. *Streptomyces* species are prolific producers of secondary metabolites, and several studies have recently reported novel anti-cryptococcal compounds from actinobacteria [45,46,47].

Among the *Streptomyces* extracts used in this study, LDB17 exhibited the strongest and most comprehensive antifungal activity, especially against *Cryptococcus* strains, including fluconazole-resistant *C. neoformans*. While LAP3 and LAP11 exhibited selective activity, particularly against *C. gattii* and reference strain *C. neoformans* H99, LDB2 and LDB11 also showed strong inhibitory effects, especially against *C. neoformans* H99.

The potent inhibitory effects of LAP3, LAP11, LDB2, and LDB11 against *C. neoformans* and *C. gattii* suggest that these *Streptomyces* strains produce bioactive metabolites with significant antifungal potential. This is especially important in light of the global burden of cryptococcosis, particularly among immunocompromised individuals such as those with HIV/AIDS or undergoing immunosuppressive therapy [48]. Current treatments like amphotericin B and fluconazole are limited by toxicity, resistance, and access issues in low-resource settings [49].

The species-specific and strain-specific inhibitory profiles observed here align with emerging evidence that *Cryptococcus* strains differ in susceptibility due to genetic diversity, capsule dimension, melanin production, and urease activity [50,51]. The more substantial effect on H99 and NIH198 compared to strain 89-610 may reflect such differences. Further studies, including metabolomic profiling and compound purification, must identify the active constituents responsible for these effects.

*Candida albicans* showed high resistance to most extracts, with no growth inhibition observed from LAP1, LAP2, LAP3, LAP8, LDB2, LDB11, or LP11. Only LDB18 demonstrated an inhibitory effect, reducing the growth by 23%. This aligns with previous findings that *C. albicans*, due to its robust biofilm formation and efflux pump activity, often exhibits resistance to many natural and synthetic antifungals [52,53,54,55]. The activity of LDB17 against *C. albicans* indicates it may possess unique bioactive constituents worthy of further exploration for antifungal drug development.

LAP1 showed the most potent inhibition against *C. glabrata* (89%), though it was nearly inactive against *C. parapsilosis* (7%). Similarly, LAP2 and LAP3 were highly effective against *C. glabrata* (77% and 86%, respectively), with modest inhibition of *C. parapsilosis* (17% and 34%, respectively). LAP8 and LAP11 inhibited *C. glabrata* by 81% and 75%, respectively, but had little to no activity against *C. parapsilosis* (0% and 14%). These results suggest that LAP extracts, particularly LAP3, contain bioactive compounds with strong specificity toward *C. glabrata*, while exhibiting limited efficacy against *C. parapsilosis*, a more resistant *Candida* species. This selectivity may be due to differences in cell wall composition or membrane sterol content between the two species [53,56,57]. The low activity against *C. parapsilosis* aligns with previous reports of its intrinsic resistance mechanisms, such as biofilm formation and efflux pumps [58].

In contrast, the LDB extracts demonstrated broader antifungal activity. LDB2 and LDB11 inhibited *C. glabrata* by 85% and 83%, respectively, and also reduced the growth of *C. parapsilosis* by 18% and 33%. LDB11 was particularly notable for its dual activity and is among the most promising extracts for further investigation. LDB18, although showing lower inhibition against *C. glabrata* (38%), exhibited the highest inhibition of *C. parapsilosis* (51%) among all extracts tested, suggesting it contains unique compounds potentially effective against *Candida* species. The observed antifungal profiles support continued screening of LDB strains for potent and potentially synergistic agents, particularly in the face of increasing antifungal resistance among *Candida* species [55].

Our data indicate the relevance of exploring the Cerrado actinobacteria for antimicrobial compound screening. We now intend to establish cooperation with research groups working on extract fractioning, biocompound identification, and drug design.

The ability to modulate the mammal immune system to enhance the elimination of the pathogen is also an essential element to be explored in the bioprospecting of novel compounds with microbicidal activity. In addition, the bioprospecting of new immunomodulators can, in the future, improve clinical immune therapy and reduce drug side effects.

The *Streptomyces*-derived extracts obtained in this study presented immunomodulation properties. TNF-α, produced by immune cells such as macrophages, natural killers (NKs), lymphocytes, mast cells, and B and T lymphocytes (Th1), is a pro-inflammatory cytokine. It mediates both innate and adaptive immunity. Previous studies have linked the modulation of TNF-α to compounds isolated from actinobacteria. Reijke and colleagues [58] showed the increase in TNF-α, among other cytokines, by in vivo administration of the immunostimulant rubratin, from *Nocardia ruber,* in patients with superficial bladder cancer. In contrast, Lee et al. [59] described a decreased cytokine production in RAW264.7 macrophages stimulated with LPS and treated with griseusrazine A, isolated from *Streptomyces griseus*. A few other studies have also reported decreased TNF-α production when mammal cells were exposed to *Streptomyces* metabolites [14,60]. In the present study, the *S. roseogriseus* LAP11 and *S. roseolus* LAP1 extracts induced spontaneous TNF-α secretion, while the *S. cellulosae* LDB11 extract suppressed their production promoted by LPS (Figure 4). The *S. cellulosae* LDB11 extract reduced the production of this cytokine even in H99-infected BMDMs (Figure 7). Thus, *S. roseolus* LAP1 and *S. roseogriseus* LAP11 presented pro-inflammatory and LDB11 anti-inflammatory action.

TNF-α, in combination with IFN-γ, increases the microbicidal activity of macrophages [61]. Nitric oxide is an intercellular messenger with a versatile role in the immune system. Activated macrophages release different effector molecules to inhibit the replication of the infectious agent, including nitric oxide. Thus, NO is considered a microbicidal mediator. Under abnormal conditions, NO overproduction leads to an anti-inflammatory response, significantly decreasing the cellular response [62]. In our work, LAP8, LAP3, and LDB2 emerged as strong NO inducers, indicating potential macrophage-activating properties (Figure 5). None of these extracts impacted the TNF-α production (Figure 4). Furthermore, although LAP8 and LDB2 extracts induced NO production, this did not affect the microbicidal activity of BMDMs (Figure 6). The NO is produced during the murine model of *C. neoformans* infection [63]. However, the capsule and melanin protect the fungi from NO microbicidal activity. *S. gancidicus* LAP2 and *S. roseogriseus* LAP11 extracts also modulated NO production, lightly stimulating BMDMs not activated by LPS and IFN-γ. We infer that the extracts *S. gancidicus* LAP2, *S. luteogriseulus* LAP3, *S. graminearus* LAP8, *S. roseogriseus* LAP11, *S. cellulosae* LDB2, and *S. tubercidicus* LDB17 modulate the expression of the gene or the activity of the nitric oxide synthase (NOS) enzyme in BMDMs. These results correlate with previous descriptions of the modulation of nitric oxide by compounds derived from *Streptomyces* sp. Lee and co-workers [59] showed the suppression of the NO production and a decrease in the expression of the NOS2 gene in macrophages exposed to griseusrazine A, from *S. griseus*. The same NO production is observed when BMDMs, activated or not by LPS + IFN-γ, were exposed to the *S. tubercidicus* LDB17 extract. Future analyses shall focus on NOS gene expression in BMDMs under exposure to these extracts.

Phagocytic cells are critical in combating infectious agents during all inflammatory processes. In addition to phagocytosis, these cells are responsible for antigen presentation and immunomodulation by releasing cytokines and growth factors [64,65]. By analyzing the influence of actinobacteria extracts on the microbicidal capacity of BMDMs in vitro, we observed that the *S. gancidicus* LAP2 and *S. cellulosae* LDB11 extracts impaired the function of macrophages against *C. neoformans* H99 (Figure 6): BMDMs presented a decreased ability to kill the yeast cells, which could then replicate inside them. Inside phagocytic cells, microorganisms must face the low availability of nutrients, the action of oxygen (ROS) or nitrogen (RNS) reactive species, and hydrolytic enzymes, in an acidic pH [66]. We speculate that the *S. gancidicus* LAP2 and *S. cellulosae* LDB11 extracts somehow affected the pathways, generating adverse conditions and provoking an anti-inflammatory profile in BMDMs. This result corroborates the previous observation of a strong suppression of the TNF-α production induced by LPS in the presence of the *S. cellulosae* LDB11 extract (Figure 4).

The IL-6 cytokine, generally considered pro-inflammatory, is a stimulating factor for B cells, leading to the production of IgGs. This cytokine is essential in the immune response against pathogens, inflammatory processes, and cell growth modulation. Interleukin-6 modulates T lymphocyte resistance to apoptosis, activates T cells, and controls the balance between Th17 cells and regulatory T cells (Treg) [67]. In this study, IL-6 and IL-10 modulation by the extracts supports their varied immunological potential. Specifically, LDB11 showed a consistent profile of anti-inflammatory cytokine induction, reinforcing its potential as a lead candidate for further studies. These results suggest promising avenues for both antifungal and immunomodulatory applications (Figure 8). Studies with mice demonstrated that an anti-inflammatory activity for IL-6 is mediated by the classical signaling pathway (activation by IL-6 receptors linked to the membrane, mbIL-6R) [68]. Because of the above, it is possible that the increase in the IL-6 production, observed in the presence of the extracts that reduced the TNF-α production and/or the microbicidal activity of infected macrophages, results from an anti-inflammatory action of *S. gancidicus* LAP2 and *S. cellulosae* LDB11 compounds.

IL-10 plays a double-edged sword role in fungal infections. On the one side, it reduces tissue damage induced by hyperinflammation and enhances immune regulation, which is helpful in systemic or chronic infections. On the other side, high levels of IL-10 can weaken the host’s antifungal immune response by inhibiting pro-inflammatory cytokine production and macrophage activation, and facilitating ongoing fungal growth [69]. In this study, induction of IL-10 production was observed in LPS-activated BMDMs infected by *C. neoformans* H99 and exposed to *S. gancidicus* LAP2, *S. cellulosae* LDB2, and *S. cellulosae* LDB11 extracts (Figure 9). Corroborating this hypothesis, these results suggest that IL-10 induction by the extracts may reduce harmful inflammation but could also hinder fungal clearance, as elevated IL-10 levels are known to promote cryptococcal persistence. Therefore, the IL-10-modulating activity of these *Streptomyces* extracts should be further investigated in vivo to clarify their overall effect on infection outcomes.

It is crucial to note that although LPS and IFN-γ were used to elicit macrophage responses in this study, reference immunomodulatory compounds such as dexamethasone or curcumin were not included. The addition of a control would offer a point of reference for determining the magnitude and direction of the induced immunomodulatory effects elicited by the *Streptomyces*-derived extracts. We recognize this to be a limitation of the present investigation and suggest its implementation in future experimental studies to make results more comparable and analysis more robust.

This work achieved the purification of actinobacteria from the rhizosphere of *S. lycocarpum* (lobeira) in the Cerrado biome and the evaluation of the derived crude extracts’ antifungal and immunomodulating properties. Our results indicate the inhibition of the growth of yeast opportunistic pathogens and the suppression or stimulation of immunomodulation on BMDMs in vitro. This work opens many research opportunities centered on screening new antifungal and/or immunomodulatory biocompounds. However, additional studies are needed before these data can be extrapolated to humans. We recognize that further experiments, such as compound characterization, mechanisms of action, cytotoxicity, genotoxicity, and in vivo studies, are required to translate these findings into drug development.

## 4. Materials and Methods

### 4.1. Soil Sampling and Actinobacteria Isolation

Soil samples were collected from a 10 cm depth of the rhizosphere of lobeira trees (*S. lycocarpum*) from diverse locations in the Federal District, Brazil, under the National System of Management of the Genetic Patrimony and Associated Traditional Knowledge (SisGEN), registration number AF34E10.

One g of soil was oven-dried at 30 °C for 24 h. The samples were then suspended in 9 mL of sterile saline solution (0.9%) and vortexed for 2 min. After 30 min of decantation, five serial 1:10 dilutions were made for each sample, and 200 µL of the 10^−4^ and 10^−5^ dilutions were plated onto plates of Asparagine Glycerol Agar (AGA) medium (1% glycerol, 0.1% L-asparagine, 0.1% K_2_HPO_4_, 0.01% actinomycete trace salt solution, and 1.5% agar), also known as International Streptomyces Project (ISP 5) medium #5. The plates were incubated at 28 °C for two weeks and observed daily.

Colonies exhibiting an actinobacteria-like appearance were streaked to single colonies on new AGA medium plates supplemented with nalidixic acid (20 µg·mL^−1^) and incubated at 28 °C. The purity of actinobacteria cultures was confirmed under a Carl Zeiss Axioskop 20 EL-Einsatz 451,487 Binocular Transmitted Light Microscope (Carl Zeiss, Oberkochen, Germany) and preserved at −80 °C in 30% (*v*/*v*) of glycerol. Morphological characterization and microscopic features were recorded. The isolates were named according to the location of the sample collection.

### 4.2. DNA Extraction, Amplification, and Sequencing of the 16s rRNA Gene

Spores from the pure actinobacteria isolates were inoculated in 3 mL of NDB (0.3% beef extract, 0.5% peptone, and 1% dextrose) and incubated at 150 rpm, at 28 °C for 2 days. Mycelial DNA was extracted using the CTAB-SDS method [70]. The full protocols for DNA extraction and PCR amplification are provided in Appendix A, Method M1.

### 4.3. Actinobacteria Identification and Phylogenetic Analysis

The quality of the obtained 16s rRNA gene sequences was analyzed with PHRED (http://asparagin.cenargen.embrapa.br/phph/ (accessed on 5 May 2021)) and Chromas tools (Technelysium Pty Ltd., South Brisbane, QLD, Australia). After editing in the program BioEdit 7.0.5.3, the sequences obtained with the two pairs of primers were combined into a consensus sequence using DNASTAR Lasergene SeqMan pro (v. 7.1.0) software. To identify the genus of the isolated actinobacteria, these sequences were compared to the GenBank database (NCBI) using BLASTN (v5). Reference sequences obtained from the database were aligned to our consensus sequences using the ClustalW multiple alignment tool available in the MEGA X 10.2.2 software and used to perform a phylogenetic analysis using the maximum likelihood method with the General Time Reversible (GTR) model and 1000 replicates for bootstrap analysis. *Bacillus subtilis* IAM 12,118 was used as an outgroup.

While the 16S rRNA gene is popular for the identification of bacteria, sequence similarity less than 99.0% is usually not regarded as adequate for species-level classification within the genus *Streptomyces*. Hence, identifications with similarity levels less than this, e.g., 98.85%, in the case of this study, were cautiously interpreted and mainly utilized to imply phylogenetic association but not definitive species assignments.

### 4.4. Preparation of Actinobacteria Crude Extracts

The crude extract from actinobacteria was prepared by following a previously established protocol [47]. The actinobacteria isolates were grown on solid AGA medium for 7 days at 28° C for sporulation. Spores were inoculated in seed medium for actinobacteria (1% starch, 0.5% glucose, 0.5% yeast extract, 0.5% K_2_HPO_4_, and 0.05% MgSO_4_.7H_2_O) and incubated at 28 °C for 3 d at 150 rpm. These cultures were used for inoculum of 15% (*v*/*v*) in 100 mL of SCN fermentation medium for actinobacteria (1% starch, 0.03% casein, 0.2% KNO_3_, 0.2% NaCl, 0.2% K_2_HPO_4_, 0.005% MgSO_4_, 0.002% CaCO_3_, and 0.001% FeSO_4_ 7H_2_O), and incubated at 28 °C for 11 days, 120 rpm, in the dark. Subsequently, the cultures were centrifuged at 10,000 g for 10 min to remove the biomass. The crude extracts were subjected to lyophilization (Liotop^®^ L101, Liobras, São Carlos, SP, Brazil), and the powder was dissolved in MilliQ water at 10% of the initial volume. The lyophilized extracts were sterilized in 0.45 µm pore size syringe filters (Kasvi) and stored at 4 °C [47].

### 4.5. Anti-Yeast Activity of the Actinobacteria Crude Extracts

Antifungal activity of the crude extracts was tested against *Cryptococcus neoformans* H99, *C. neoformans* 89-610 (fluconazole-resistant strain), *C. gattii* NIH198 (generously provided by Dr Joseph Heitman Laboratory, Duke University Medical Center, Durham, NC, USA), and also against *Candida albicans*, *C. glabrata*, and *C. parapsilosis* (kindly provided by Dr. Patricia Albuquerque, University of Brasilia, Brazil). Growth inhibition tests were performed according to the CLSI M27-A3 protocol [71]. The fungal strains were grown in Sabouraud Dextrose broth (SDB) (1% peptone, 2% glucose) and incubated at 30 °C for 20 h. The appropriate dilutions were made so that the final inoculum corresponded to a concentration between 1 × 10^4^ and 2 × 10^4^ CFU mL^−1^. The experiments were performed in RPMI-1640 medium buffered with MOPS (0.165 mol/L) and supplemented with 0.2% glucose in 96-well microplates, with a total volume of 200 µL per well, and incubated at 37° C for 72 h without shaking. The optical density of the growth was observed at 530 nm. An initial reading was taken before incubation to deduce the final absorbance values and exclude biases, such as the pigmentation of the extract.

As previously reported by our group [72], fluconazole (Flu) and sodium butyrate (NaBut) were used as control drugs at the minimum concentration of inhibition.

### 4.6. Animals and Ethics Statement

*Mus musculus* C57BL/6 male mice, aged 8 to 12 weeks, were raised in the Institute of Biological Sciences, University of Brasilia, under appropriate conditions with water and “ad libitum” feed. The Committee on Ethics in Animal Use (CEUA-UnB) approved the experimental procedures (project no.23106.113772/2020-80), considering the protocols to reduce pain, suffering, and distress. The experiments were conducted following the protocols of the National Council for the Control of Animal Experiments (CONCEA). There is no humane endpoint in this study, only one point to euthanize the mice. No exclusion criterion was used to compose the groups or conduct the experiments.

### 4.7. Murine Cell Culture

Bone marrow-derived macrophages (BMDMs) were obtained by differentiating murine hematopoietic stem cells according to the protocol of Lutz et al. [73] and standardized by our group and approved by CEUA-UnB from a pool of hematopoietic cells. Euthanized mice femur and tibia obtained from 4 mice were flushed to obtain bone marrow cells. Erythrocytes were lysed using the lysis solution (8.3 g L^−1^ NH_4_Cl; 0.01 M Tris-HCl buffer). Hematopoietic stem cells (2 × 10^6^) were incubated in RPMI-1640 medium with gentamicin, supplemented with 10% fetal bovine serum (FBS) (Gibco, Grand Island, NY, USA), 20 ng mL^−1^ granulocyte–macrophage stimulating factor (GM-CSF) (ImmunoTools, Friesoythe, Germany), and 50 µM β-mercaptoethanol (Sigma-Aldrich, St. Louis, MO, USA) at 37 °C under a 5% CO_2_ atmosphere. After 6 days of incubation, the cell culture medium, supplemented as above, was replaced. After 8 days of culture, the dendritic cells were removed from the culture supernatant, and the M1-like macrophages, adhered to the plates, were stripped with TrypLE solution for 30 min.

Since the complete polarization to M1 macrophages during the differentiation is not assured, even when employing GM-CSF, and because additional analyses of important M1 markers were not performed, macrophages were assumed to have a profile similar to M1, i.e., M1-like macrophages.

### 4.8. Cytotoxicity Evaluation of the Actinobacteria Extracts on BMDMs

The cytotoxicity of the actinobacteria extracts was evaluated using the CytoTox 96^®^ Non-Radioactive Cytotoxicity assay kit (Promega, Madison, WI, USA) according to the manufacturer’s protocol. The highest concentration initially stipulated for the cytotoxic evaluation of the extracts to BMDMs was 10% of the final volume, as this is the sample concentration generally assessed by our group in immunological tests. Lower final concentrations of 7% and 5% extracts were also studied. A final concentration of 1 or 0.5% was employed for the extracts that demonstrated cytotoxicity at 5%. The concentration of 5% was used for extracts displaying cell viability near or equal to 70%.

### 4.9. In Vitro BMDM Activation Assays

The BMDMs were separated as Lutz et al. [73] recommended, and a cell density of 1 × 10^5^ cells per well was used in all experiments. The cells were incubated with different extracts for 24 h in the following groups: (i) cells + extract; (ii) cells + extract + lipopolysaccharide (LPS); and (iii) cells + extract + LPS + interferon-γ (IFN-γ). The final concentrations were as follows: LPS 0.5 μg/mL; IFN-γ 20 μg/mL. A PBS control group was employed in each experiment. The cell supernatants were collected after incubation and kept at –20 °C.

### 4.10. Cytokine Quantification

The sandwich ELISA method was used to determine the levels of tumor necrosis factor (TNF-α), interleukin-6 (IL-6), and interleukin-10 (IL-10). TNF-α Mouse Uncoated, IL-6 Mouse Uncoated, and Mouse IL-10 Uncoated ELISA kits (Invitrogen, Carlsbad, CA, USA) were employed according to the manufacturer’s instructions. The quantification of cytokines was also performed with the supernatant of BMDMs exposed to the extracts and of BMDMs infected with *C. neoformans* H99 and then exposed to the extracts.

### 4.11. Nitric Oxide Production by BMDMs Exposed to Actinobacteria Extracts

The production of nitric oxide (NO) by BMDMs was measured according to Bryan & Grisham [74]. The Griess solution was incubated with the supernatants of the different experimental groups for 15 min in the dark. The absorbance was measured in a spectrophotometer at a wavelength of 540 nm after the reaction was stopped. A nitrite standard curve was used to compare and quantify NO in the samples.

### 4.12. Interaction Assay of BMDMs and C. Neoformans H99 Yeast Cells

BMDMs were inoculated into 96-well plates at a density of 1 × 10^5^ cells per well. Experimental groups were established with or without LPS activation (0.5 µg/mL) and incubated for 3 h at 37 °C under an atmosphere of 5% CO_2_. Yeasts were opsonized with the monoclonal antibody 18 B7 (39 µg/mL), kindly provided by Professor Arturo Casadevall (Department of Molecular Microbiology and Immunology, Johns Hopkins Bloomberg School of Public Health, Maryland, USA), for 30 min before infection. Yeast cells were then added to the BMDM cultures at a multiplicity of infection (MOI) of 5 and incubated for 2 h. Subsequently, the wells were washed with RPMI-1640 medium at 37 °C to remove non-internalized yeast cells. Replacement of the medium was carried out, with LPS re-stimulation carried out for the appropriate group, and the addition of extracts at the described concentrations. Only sterile PBS was added to the 2 h infection group as an internalization control.

After 24 h of incubation, the supernatants were collected and stored at −20 °C. The cultures were washed with PBS to remove free yeast cells [75,76].

### 4.13. C. neoformans H99 Colony-Forming Unit Quantification

BMDMs from the 2 h or 24 h interaction assay groups were lysed with a 0.05% SDS solution. An amount of 100 µL of the solution was added to each triplicate well and diluted 1:10 after resuspension. An amount of 50 µL of the suspension was plated on Sabouraud Dextrose Agar medium (SDA; 1% peptone, 2% glucose, and 1.2% agar) and incubated at 28 °C. After three days, the colony-forming units (CFUs) were counted.

### 4.14. Statistical Analysis

Data analysis was performed using GraphPad Prism 8.0 software. One-way ANOVA with multiple comparisons was applied when comparing within groups, and a *t*-test was used when analyzing between groups. The statistical difference for analysis within groups was demonstrated with * or # for *p* < 0.05; ** or ## for *p* < 0.01; *** or ### for *p* < 0.001; and **** or #### for *p* < 0.0001. For significant differences in the analyses between groups, the same *p* values were considered and indicated by the symbol “#” followed by a slash.

## 5. Conclusions

This study presented the antifungal and immunomodulatory properties *of Streptomyces* spp. isolated from the Cerrado rhizospheric soil. *Streptomyces* spp. isolates LAP2, LAP8, LDB2, and LDB11 demonstrated significant antifungal activity against yeasts with lower cytotoxicity than other isolates. *Streptomyces* sp. LDB17 exhibited the highest antifungal efficacy, but also higher cytotoxicity, which limited its further use in immunological assays. Extracts of isolates LAP2, LAP8, and LDB2 promoted pro-inflammatory cytokine production, whereas LDB11 demonstrated a potential immunosuppressive effect by suppressing TNF-α levels and IL-10 production.

These findings suggest that *Streptomyces* spp. yield bioactive compounds with dual applications as antifungal and immunomodulatory agents. Further work is needed, such as metabolomic profiling and extract fractionation, to identify and characterize bioactive compounds present in the extract with both antifungal and immunomodulatory activity. This research highlights the potential of *Streptomyces* spp. as sources of novel antifungal agents with immunomodulatory properties.

## Figures and Tables

**Table 1:** Macroscopic features of actinobacteria isolates.

Identification	Isolates	Micellium Pigments		Soluble Pigments
		Substrate	Aerial/Spores	
LAP	1	Light brown	Yellow	Orange
	2	Brown	Light brown	Orange
	3	Light brown	Light brown	Black
	8	Light pink	Pink/gray	Orange
	11	Light brown	Light gray	Wine red
LDB	2	Brown	Yellow	Brown
	11	Brown	Light gray	Orange
	17	–	–	Yellow
	32	Dark blue	Light blue/gray	–
	34	Pink	Light pink	–
LS	1	Light brown	–	Orange
	2	Light yellow	–	Orange
	3	–	–	Yellow

## Data Availability

Data presented in this study is contained within the article. Further inquiries can be directed to the corresponding author.

## Appendix A. Method M1: Methods for DNA Extraction, Amplification, and Sequencing of the 16s rRNA Gene

Spores from the pure actinobacteria isolates were inoculated in 3 mL of NDB (0.3% beef extract, 0.5% peptone, and 1% dextrose) and incubated at 150 rpm, at 28 °C, for 2 days. Mycelial DNA was extracted using the CTAB-SDS method [70]. Actinobacteria cultures were collected and washed 3× with TE buffer (1 M Tris-Cl, 0.5 M EDTA, pH 8.0). The material was macerated with porcelain grail and pistil. The cells were lysed by vortexing after the addition of TE, CTAB-NaCl (2%), NaCl (1.3 M), lysozyme (0.2 mg/mL), proteinase K (0.2 mg/mL), and sodium dodecyl sulfate (SDS) (0.35%). Mixtures were incubated at 37 °C for 1 h and then at 65 °C for 15 min. The aqueous phase was treated with RNase A (10 mg/mL) for 45 min at 37 °C. The samples were treated twice with a Phenol–Chloroform–Isoamyl Alcohol (25:24:1) solution, followed by the addition of a Chloroform–Isopropanol (24:1) solution. For DNA precipitation, sodium acetate (0.3 M) and isopropanol (0.6 *v*/*v*) were added, and the samples were centrifuged at 13,000 rpm for 10 min. The DNA pellet was washed twice with 70% ethanol and resuspended in sterile MilliQ water. The samples were kept at −20 °C.

The genomic DNA was amplified using universal primers for 16S rDNA, 27f (5′-GAGTTTGATCMTGGCTCAG-3′) and 1492r (5′-ACGGYTACCTTGTTACGACTT-3′) [77], and actinobacteria-specific 16S rDNA primers, SC-Act-235aS20 (5′-CGCGGCCTATCAGCTTGTTG-3′) and SC-Act-878aA19 (5′-CCGTACTCCCCAGGCGGGG-3′) [78]. Reactions were performed in a final volume of 25 µL, containing 1× Taq buffer, 2 mM MgCl_2_, 800 µM dNTP mix, 0.4 pmol of each primer, 1.2 U Taq DNA polymerase (Invitrogen), and 1 µL of DNA as template. An initial denaturation at 95 °C for 3 min was followed by 35 cycles of 95 °C for 30 s; 52 °C (universal primers) or 70 °C (actinobacteria-specific primers) for 60 s; and 72 °C for 1.5 min (universal primer) or 72 °C for 60 s (actinobacteria-specific primers) with a final extension at 72 °C for 10 min. The PCR products were analyzed by electrophoresis on a 1.2% agarose gel containing 0.5 µg L^−1^ ethidium bromide by comparison with the 1kb molecular marker (Kasvi).

The PCR products were treated with the SAP (0.18 U/µL) and EXOI (0.2 U/µL) enzymes at 37 °C for 1 h 30 min, followed by 80 °C for 20 min and 4 °C for 20 min. The samples were sent for sequencing in a final volume of 7 µL, containing 3 µL of primer, and in a final DNA concentration between 5 and 20 ng at the DNA Sequencing Laboratory of the Catholic University of Brasilia (UCB), using the Genetic Analyzer 3130 (Applied Biosystems) sequencer.
